# Laparoscopic Single Anastomosis Sleeve Ileal Bypass Versus Laparoscopic Roux-en-Y Gastric Bypass as Single Stage Procedure for Management of Patients with Class V Obesity (BMI ≥ 60 kg/m^2^): Short-Term Follow-Up

**DOI:** 10.1007/s11695-025-07930-5

**Published:** 2025-05-28

**Authors:** Mohamed Wael, Mohamed Mosaad Kandel, Hashem Altabbaa, Mostafa Refaie Elkeleny

**Affiliations:** 1 Alexandria Main University Hospital, Alexandria, Egypt; 2 Alexandria University, Alexandria, Egypt; 3 Port Said University, Port said, Egypt

**Keywords:** Extreme obesity, Class V obesity, Obesity-related comorbidities, RYGB, SASI

## Abstract

**Background:**

Individuals with clinically severe obesity and a BMI ≥ 60 kg/m^2^ (class V obesity) have a higher incidence of both obesity-related comorbidities and anticipated operative difficulty, with a subsequent higher risk of perioperative morbidity and mortality and a longer hospital stay. In patients with class V obesity, the definitive bariatric procedure is still a matter of debate. This study compared surgical procedures (SASI vs. RYGB) in people with class V obesity. The primary objective was to compare weight loss after both procedures over a 1-year follow-up. Secondary outcomes included the evaluation of the incidence of the operative time (skin-to-skin), postoperative complications, duration of hospital stay, rate of conversion to the open technique, and quality of life, as well as amelioration of obesity-related comorbidities.

**Methods:**

From January 2019 to December 2022, the data of 73 consecutive patients with class V obesity was collected, who underwent either standard RYGB (*n* = 40) or SASI (*n* = 33) at the General Surgery Department of Alexandria University Hospital and some non-governmental hospitals.

**Results:**

There was no statistically significant difference between both groups as regards mean age (p = 0.012), sex (*p* = 0.250), preoperative BMI (*p* = 0.754), or preoperative incidence of obesity-related co-morbidities. The SASI procedure showed a statistically shorter operative time (*p* < 0.001). There was no significant difference between the two groups as regards the incidence of postoperative surgical complications, either early cmplications (21.1% and 20% in both SASI and RYGB, respectively, *p* = 0.770) or late (beyond 30 days) complications (15.2% and 15% in SASI and RYGB, respectively, *p* = 1.000), with neither conversion nor intra-operative mortality in both groups. However, the SASI group showed a significant shorter postoperative hospital stay (*p* < 0.001). During the follow-up period, both operations demonstrated a significant overall resolution of pre-operative obesity-related comorbidities, a significant increase in postprandial 6 weeks postoperative GLP-1 with statistically more rise in the SASI group in the postprandial GLP-1 compared to the RYGB group (*p* < 0.001). There was no mortalities in both group during the follow up duration.

**Conclusion:**

In patients with class V obesity, the SASI procedure had a statistically shorter operative time (skin-to-skin) and a shorter hospital stay compared to RYGB. Both procedures resulted in satisfactory weight loss, as well as comparable improvements in obesity-related comorbidities.

## Introduction

Obesity is labeled by the World Health Organization (WHO) as the most obviously visible, but unfortunately, the most neglected worldwide public health problem [1]. Obesity is associated with numerous obesity-related comorbidities, including hypertension (HTN), diabetes mellitus (DM), cardiovascular diseases, dyslipidemia (DL), and some malignancies [2]. Up to date, bariatric surgery was superior to conservative treatment in the management of both weight excess and obesity-related comorbidities in terms of outcome and health expenses [3].

Performing bariatric surgery on class V obese patients has multiple challenges. Technically, this population typically requires longer laparoscopic instruments due to anticipated operative difficulties. Male patients with higher grades of obesity often need longer instruments than female patients with the same obesity level because they have more visceral fat, a bigger liver, and require more torque to hold the instruments [4, 5].

Medically, these patients usually have multiple comorbidities, making the perioperative care for a bariatric surgery difficult and resulting in many surgical complications, such as insufficient cardiopulmonary reserve, acute respiratory failure, recurrent chest infections, and deep vein thrombosis [6, 7].

Laparoscopic Roux-en-Y gastric bypass (RYGB) is still one of the most common bariatric and metabolic surgeries in the world. Since its initial introduction, the procedure has been subjected to several technical modifications aiming to reach both more weight loss and minimal nutritional complications. The RYGB procedure divides the small residual gastric pouch on the lesser stomach curvature (15–30 mL) completely from the gastric remnant and then anastomoses it to the jejunum bowel loops, resulting in an alimentary (or Roux limb) of typically 1–1.5 m. An entero-enteric anastomosis between the alimentary limb and the excluded biliopancreatic limb then restores bowel continuity [8].

Bipartition is based on the theory of digestive adaptation [9]. Azevedo, et al. [9] talked about its’ long-term data. They suggested bipartition to increase nutrient stimulation in the distal gut and decrease nutrient stimulation in the proximal small intestine without deactivating the duodenum and jejunum. The SASI procedure is similar to a sleeve gastrectomy operation with transit bipartition, but it uses a single anastomosis instead of a Roux-en-Y technique. Early reports are encouraging about its safety and effectiveness [10–12].

Several studies showed that the SASI bypass is effective and comparable to RYGB in terms of weight loss and metabolic control, and it has the advantage of not producing excluded segments and reducing nutritional deficiencies [13–15].

However, in the population with class V obesity, the best bariatric procedure is still debatable. This study compared both surgical operations (SASI vs. RYGB) in class V obese patients. The primary objective was to compare weight loss after both procedures over a 1-year follow-up. Secondary outcomes included the operative time (skin to skin), postoperative complications, duration of hospital stay, rate of conversion to the open technique, improvements in the quality of life, amelioration of obesity-related comorbidities, and change in the level of GLP-1 6 weeks postoperative.

## Methods

### Study Design

This retrospective study analyzed the records of 73 consecutive class V obese patients who underwent either standard RYGB procedure (*n* = 40) or SASI procedure (*n* = 33) from January 2019 to December 2022 at the General Surgery Department of Alexandria University Hospital and some non-governmental hospitals.The retrospective nature of the study eliminated the need of ethical patient approval. Institutional ethics review board approval was obtained prior to data collection.

(1) Age ranging from 18 to 60 years; (2) BMI greater than 60 kg/m^2^ with or without related comorbidities. (3) The patient must have failed to adhere to supervised conservative management for obesity for at least 6 months. (4) Patient’s willingness to undergo prolonged follow-up sessions with the surgeon and nutritionist.

The following patients were excluded: (1) BMI less than 60 kg/m^2^; (2) severe eating disorder (binge eating); (3) patients with acute systemic infections; (4) endocrinal diseases; (5) active peptic ulcer disease; (6) patients with a general contraindication for laparoscopy; and (7) patients with a history of major abdominal operations, such as splenectomy or colectomy.

The patients were preoperatively informed that both procedures anticipate similar outcomes in terms of surplus weight loss and the resolution of obesity-related comorbidities, but a lower incidence of nutrient deficiency is anticipated after SASI bypass due to its bipartition nature. All patients were educated and screened with preoperative medical, nutritional, and psychological assessments.

### In Accordance with Our Institute Policy, Patients and Procedures Were Selected for Bariatric Surgery Based on the Following Criteria

(1) Age ranging from 18 to 60 years; (2) BMI greater than 60 kg/m^2^ with or without related comorbidities. (3) The patient must have failed to adhere to supervised conservative management for obesity for at least 6 months. (4) Patient’s willingness to undergo prolonged follow-up sessions with the surgeon and nutritionist.

The following patients were excluded: (1) BMI less than 60 kg/m^2^; (2) severe eating disorder (binge eating); (3) patients with acute systemic infections; (4) endocrinal diseases; (5) active peptic ulcer disease; (6) patients with a general contraindication for laparoscopy; and (7) patients with a history of major abdominal operations, such as splenectomy or colectomy.

The patients were preoperatively informed that both procedures anticipate similar outcomes in terms of surplus weight loss and the resolution of obesity-related comorbidities, but a lower incidence of nutrient deficiency is anticipated after SASI bypass due to its bipartition nature. All patients were educated and screened with preoperative medical, nutritional, and psychological assessments.

### Surgical Technique

A fixed bariatric surgical team, composed of an expert main bariatric surgeon with two bariatric surgeon assistants, performed all procedures under general anesthesia in the french operative position.

- The RYGB was performed in an antecolic fashion. Gastric pouch was about 30–50 cc. Then a biliopancreati-c limb, approximately 50–100 cm, was measured starting from the ligament of Treitz. The roux limb was measured 150 cm from the jejunal division point. The mesentric defects (Petersen and jujenal defects) were closed routinly.
- The SASI operation began with performing a wide sleeve gastrectomy, then the selected intestinal loop (250–300 cm proximal to the ileocecal junction) was brought up to the gastric sleeve. A linear cutting stapler was used to perform an isoperistaltic side-to-side anastomosis to the anterior wall of the stomach antrum (2 cm from the pylorus). The anastomosis diameter did not exceed four centimeters. Then, the anterior wall of the gastroileal anastomosis was closed by running sutures using V-Lock 2/0.

### Postoperative Care of Both Groups

All patients were kept NPO (nothing per mouth) until they underwent a Gastografin swallow X-ray study to rule out leaks. If there were no leaks, patients began taking oral fluids immediately after the test on postoperative day 2. If there were no complications, the patients were discharged on a liquid diet for 2 weeks, and the drain was removed before discharge.

Nutritionists monitored the patients’ diet progression. (A) Weeks 1–4: full liquids or pure solids, such as mashed potatoes and applesauce. (B) Week 5: soft foods, such as rice and overcooked vegetables. (C) Week 6: regular foods, such as chicken and bread, should be consumed. (D) Week 7: more regular foods. Exercise involved walking for 30 min daily and participating in normal daily activities as tolerated. Upon the nutritionist’s prescription, all patients received oral multivitamins, calcium, and iron supplements starting from the second postoperative week until they resumed regular food intake (with follow-up laboratory investigations withdrawn on demand according to the patient’s clinical status).

#### Definitions and Study Endpoints

The primary endpoint of the study was comparing the excess weight loss after both procedures, while the secondary endpoint was comparing postoperative complications, skin-to-skin operative time, length of hospital stay, and related comorbidities improvement after both procedures.

The study endpoints were defined according to the standardized outcomes reporting in metabolic and bariatric surgery [16] as follows:

- Percentage of total weight loss (%TWL): [(preoperative weight − final weight)/preoperative weight] × 100.
- Percentage of excess BMI loss (%EBMIL): [(initial BMI – final BMI)/(initial BMI-25)] × 100.
- Complete remission of DM: fasting plasma glucose level < 100 mg/dL or HbA1 C level < 6% without further use of hypoglycemic medication at 1 year after surgery.
- Hypertension remission was considered if the patients had normal blood pressure (BP <120/80) off antihypertensive treatment.
- Dyslipidemia remission: normal lipid profile with no medications.
- GERD remission: absence of symptoms, no further medication use, and a normal 24-h pH study.
- Postoperative complications refer to the unintended and undesirable outcomes of the operation, which directly affect the patient.

#### Data Collection

For included patients, the following data was collected from our obesity database.

- Operative details including the average duration of the procedure, intraoperative complications (such as bleeding, organ damage, and conversion), the reason for the conversion if happened, and any additional procedures (like cholecystectomy).
- Follow-up details. All follow-up visits were scheduled for patients at 3, 6, and 12 months postoperatively, during which relevant data were collected. It included the following: (a) weight loss data; (b) laboratory investigations; (c) amelioration of obesity-related comorbidities; (d) postoperative complications; (e) quality of life using the Moorehead-Ardelt Quality of Life Questionnaire II (M-A QoLQII) [17]; and (f) postoperative serum GLP-1 after 6 weeks.

#### Statistical Analysis

The statistical analysis were conducted using IBM SPSS version 25. Quantitative variables like age, BMI, and weight using mean and median as measures of central tendency and standard deviation, minimum and maximum as measures of dispersion, and categorical variables like gender and preoperative habits were summarized. The frequency and percentage also were summarized.

A Wilcoxon-signed rank test conducted to examine the statistically significant differences in the median scores of each quality of life question before and after surgery. Also the pre- and postoperative total scores by summing the scores of the six questions for each patient before and after surgery were computed. A Mann–Whitney test or independent *t* test was done to detect a statistically significant difference in the median EWL between categories of gender, BMI, onset of obesity, etc. The abnormally distributed quantitative variables were determined by the KS test.

Categorical variables were compared with the chi-square test or Fisher’s exact test. For effect size we used Cohen’s *d* for continues data and odds/risk ratios for categorical data and their corresponding 95% confidence interval (95%CI).

A repeated measure ANOVA test used to determine whether there was a statistically significant difference in the mean weight, percentage EWL, BMI, and percentage excess BMI loss preoperatively at 6 months and at the end of the study. The Kruskal–Wallis test was used to study if there is a statistically significant difference in the median EWL between different categories of preoperative habits and age. A Friedman two-way ANOVA used to determine the statistical significance. All statistical tests were judged at 0.05 significance level.

## Results

A total of 73 class v obese consecutive patients were included in the study. Thirty-three patients were managed by the SASI procedure (group A), while 40 patients were managed by the RYGB procedure (group B).

Table 1 displays the characteristics of the entire cohort, including demographics and BMI of both study groups. In terms of mean age and sex, there was no statistically significant difference between the two groups as regards age (*d* = 0.37, 95% CI (− 0.09–0.83), *p* = 0.122), sex (OR = 2.13, 95% CI (0.67–6.76), *p* = 0.250) and preoperative BMI (*d* = 0.08, 95% CI (− 0.38–0.54), *p* = 0.754).

**Table 1:** Comparison between the studied groups according to anthropometrics

	SASI group (*n* = 33)		RYGB group (*n* = 40)		Test of Sig	OR (95%CI) *d* (95%CI)	*p*
	No	%	No	%			
**Sex**						2.13 (0.67 − 6.76)	
Male	9	27.3	6	15.0	*χ*^2^ = 1.668		^FE^*p* = 0.250
Female	24	72.7	34	85.0			
**Age (years)**							
20–30	0	0.0	2	5.0	*χ*^2^ = 10.881		^MC^*p* = 0.012
31–40	10	30.3	2	5.0			
41–50	15	45.5	2	5.0			
50–60	8	24.2	18	45.0			
Min.–Max	33.0–59.0		22.0–59.0		*t* = 1.564		0.122
Mean ± SD	45.18 ± 7.28		48.25 ± 9.12			**0.37 (**− **0.09–0.83)**	
Median	45.0		48.0				
**Weight (kg)**							
Min.–Max	150.0–170.0		156.0–180.0		0.600		0.551
Mean ± SD	164.09 ± 6.61		164.97 ± 5.98			0.14 (− 0.32–0.60)	
Median	165.0		163.5				
**Height (cm)**							
Min.–Max	155.0–164.0		150.0–170.0		0.336	0.08 (− 0.38–0.54)	0.738
Mean ± SD	160.97 ± 2.40		161.33 ± 5.66				
Median	162.0		160.0				
**BMI** (kg/m^2^)							
Min.–Max	60.94–66.41		60.21–69.33		0.315	0.08 (− 0.38–0.54)	0.754
Mean ± SD	63.31 ± 1.63		63.48 ± 2.70				
Median	62.89		63.28				
**Excess weight**							
Min.–Max	40.0–108.0		36.80–94.50		3.292	− 0.77 (− 1.25**–**0.29)	0.002
Mean ± SD	72.88 ± 20.52		59.55 ± 13.94				
Median	78.0		59.50				

*BMI* body mass index, *χ*^2^ value for chi-square, *FE* Fisher exact test, *SD* standard deviation, *OR* odds ratio, *d* Cohen’s *d* effect size, *95%CI* 95% confidence interval, *t* Student’s *t*-test

Table 2 displays the incidence of obesity-related comorbidities among the included patients. Most patients had one or more pre-existing related comorbidities. Five patients in both groups had non-obesity-related complications (one patient in the SASI group and four patients in the RYGB group). There was no significant difference between the two groups in terms of preoperative obesity-related comorbidities (OR = 0.28, 95% CI (0.03–2.65), *p* = 0.369).

**Table 2:** Comparison between the studied groups according to obesity-related comorbidities

Medical history	SASI group (*n* = 33)		RYGB group (*n* = 40)		*χ*^2^	OR (95%CI)	*p*-value
	No	%	No	%			
Patients without obesity-related comorbidities	1	3.0	4	10.0	1.377	0.28 (0.03–2.65)	^**FE**^***p* = 0.369**
Patients with obesity-related comorbidities	32	97.0	36	90.0			
HTN	20	60.6	28	70.0	0.709	1.52 (0.57–4.01)	**0.462**
Reflux	13	39.4	16	40.0	0.003	1.03 (0.4–2.63)	**1.000**
Dyslipidemia	4	12.1	8	20.0	0.817	1.81 (0.49–6.66)	^**FE**^***p* = 0.528**
OSAS	15	45.5	12	30.0	1.853	0.51 (0.19–1.35)	**0.225**
Menestrual irregularity	2	6.1	2	5.0	0.039	0.82 (0.12–6.13)	^**FE**^***p* = 1.000**
Muscloskeletal disease	5	15.2	6	15.0	0.0	0.99 (0.27–3.58)	^**FE**^***p* = 1.000**
DM	9	27.3	16	40.0	1.301	1.78 (0.66–4.80)	**0.324**
Vaicose viens	0	0.0	2	5.0	1.696	0.54 (0.43–0.67)	^**FE**^***p* = 0.498**
Bronchial asthma	2	6.1	0	0.0	2.493	^**−**^	^**FE**^***p* = 0.201**
Urinary stress incontinence	2	6.1	0	0.0	2.493	^**−**^	^**FE**^***p* = 0.201**

*HTN* hypertension, *DM* diabetes mellitus, *χ*^2^ value for chi-square, *FE* Fisher exact test, *OR* odd ratio, *95%CI* 95% confidence interval

^*^Statistically significant at *p* ≤ 0.05

The operative details were compared between both groups, as shown in Table 3. Operative time was significantly longer in the RYGB group compared to the SASI group (*r* = 0.65, 95% CI (0.48–0.82), *p* < 0.001). As regards the operative complications, intraoperative bleeding occurred in one case of the SASI group due to injury to the upper pole of the spleen, which was controlled by compression. In the SASI group, two intraoperative complications related to staplers were encountered: one of these complications was a broken stapler, which was replaced by another one, and a misfired reload, which was replaced by another reload medial to the previous one. One patient in the SASI group experienced bleeding at the trocar site, where bipolar diathermy failed to control the bleeding effectively, and multiple sutures using a fascial closure instrument ultimately achieved control. Two cases in the RYGB group suffered from bowel injuries; a superficial bowel injury happened in one case during bowel manipulation, which was repaired by a Vicryl® 3/0 suture, while a transfixing bowel injury during bowel counting occurred in another case, which was repaired by a Vicryl® 3/0 suture.

**Table 3:** Comparison between the studied groups according to operative data

Operative data	SASI group (*n* = 33)	RYGB group (*n* = 40)	*Z*	*r* (95%CI)	*p*
**Operative time (skin-to-skin) (min.)**					
Min.–Max	60.0–170.0	80.0–222.0	5.575^*^		< 0.001^*^
Mean ± SD	87.27 ± 30.29	145.20 ± 41.33		**0.65 (0.48–0.82)**	
Median	75.0	146.50			
	*N* (%)	*N* (%)	**χ**^**2**^	**OR (95%CI)**	^**FE**^***p***
**Intraoperative complications**	**3 (9.1)**	**2 (5)**	**0.474**	**0.53 (0.83–3.35)**	**0.653**
Bleeding	1 (3)	0 (0)	1.229	-	0.452
Bowel injury	0 (0)	2 (5)	1.696	-	0.498
Stapler related	2 (6.1)	0 (0)	2.493	-	0.201
Trocar site bleeding	1(3)	0(0)	1.229	-	0.452
**Conversion to Open**	**0 (0)**	**0 (0)**	**0.0**	-	**0.0**
**mortality**	**0 (0)**	**0 (0)**	**-**	^−^	^**−**^
**Associated procedures**					
**Laparoscopic cholecystectomy**	**3 (9.1)**	**4 (10)**	**0.017**	**1.11 (0.23–5.36)**	**1.000**

*Z Z* for Mann–Whitney test, *χ*^2^ chi-square test, *FE* Fisher exact test, *SD* standard deviation, *OR* odds ratio, *r* effect size suitable for Mann–Whitney *U* test, *95%CI* 95% confidence interval

^*^Statistically significant at *p* ≤ 0.05

Apart from these intraoperative complications, no other patients suffered from major intraoperative complications. In both groups, there was neither conversion to open technique nor intra-operative mortality.

Regarding the postoperative course of both groups, as shown in Table 4,

- The postoperative hospital stays were significantly longer in the RYGB group versus the SASI group (*r* = 0.44, 95% CI (0.23–0.65), *p* < 0.001).
- Postoperative complications: severity was categorized according to the Clavien-Dindo classification. Grade I: minor complications were seen in 18% of the SASI group and 22.5% of the RYGB group. Grade II: moderate complications were seen in 3% of the SASI group and 7.5% of the RYGB group. Grade III: subtype A complications were absent in the SASI group but were seen in 5% of the RYGB group. Complications of subtype B were seen in 15% of the SASI group, but they were non-existent in the RYGB group. Neither group had serious or life-threatening complications (grade IV or V).

**Table 4:** Comparison between the studied groups according to postoperative data

Postoperative data	SASI group (*n* = 33)		RYGB group (*n* = 40)		Test of Sig	OR (95%CI) *r* (95%CI)	*p*
	No	%	No	%			
**Hospital stay (days)**						**Hospital stay (days)**	
Min.–Max	2.0–5.0	2.0–7.0	Z = 3.753^*^	0.44 (0.23**–**0.65)	< 0.001^*^	Min.–Max	2.0–5.0
Mean ± SD	2.95 ± 0.87	3.78 ± 1.24				Mean ± SD	2.95 ± 0.87
Median	3.0	3.50				Median	3.0
**Complications**	12	36.3	14	35.0	χ^2^ = 0.015	0.94 (0.36**–**2.47)	1.000
**Severity** **(Clavien-Dindo classification)**					χ^2^ = 8.469		**0.037**
Grade I	6	18	9	22.5			
Grade II	1	3	3	7.5			
Grade III							
A	0	0	2	5			
B	5	15	0	0			
Grade IV Grade V	0	0	0	0			
**Early (within 30 days)**	**7**	**21.2**	**8**	**20.0**	**0.161**	**0.79 (0.25–2.53)**	**0.770**
Gastric leakage	1	3.0	1	2.5	0.019	**0.82 (0.05–13.6)**	^FE^*p* = 1.000
Intra-abdominal abscess	0	0.0	2	5.0	1.696	^−^	^FE^*p* = 0.498
Intraluminal Bleeding	1	3.0	0	0.0	1.229	^−^	^FE^*p* = 0.452
Wound seroma	2	6.1	2	5.0	0.039	**0.82 (0.11–6.12)**	^FE^*p* = 1.000
Wound infection	2	6.1	2	5.0	0.039	**0.82 (0.11–6.12)**	^FE^*p* = 1.000
Basal atelectasis	0	0.0	1	2.5	1.229	^−^	^FE^*p* = 0.452
Pulmonary embolism	1	3.0	0	0.0	1.229	^−^	^FE^*p* = 0.452
**Late (beyond 30 days)**	**5**	**15.2**	**6**	**15.0**	**0.0**	**0.99 (0.27–3.58)**	^**FE**^***p* = 1.000**
Persistent vomiting	2	6.1	0	0.0	2.493	^−^	^FE^*p* = 0.201
Portal site hernia	1	3.0	0	0.0	1.229	^−^	^FE^*p* = 0.452
Anemia	0	0.0	2	5.0	1.696	^−^	^FE^*p* = 0.498
Vitamin B_12_ deficiency	1	3.0	0	0.0	1.229	^−^	^FE^*p* = 0.452
Constipation	0	0.0	4	10.0	3.491	^−^	^FE^*p* = 0.122
Malnutrition	1	3.0	0	0.0	1.229	^−^	^FE^*p* = 0.452

*χ*^2^ chi-square test, *SD* standard deviation, *Z Z* for Mann–Whitney test, *OR* odds ratio, *r* effect size suitable for Mann–Whitney *U* test, *95%CI* 95% confidence interval

^*^Statistically significant at *p* ≤ 0.05

Postoperative complications were classified according to the onset into early (within 30 days of the operation) and late complications (beyond 30 days postoperatively).

### Early Complications

A patient in the SASI group developed stapler line leakage near the angle of His. The patient was then managed by laparoscopic drainage and endoscopic stent insertion, which was then extracted 6 weeks after complete healing was confirmed by a follow-up endoscopy, an upper contrast study, and clinical follow-up, while another patient in the RYGB group had gastric leakage almost from the gastric pouch, which was diagnosed on POD 7. This patient presented with abdominal pain. A CT abdomen revealed a retro-gastric collection of about 15 × 6 cm. The patient was then admitted to the hospital for four days under antibiotic therapy and discharged in good condition. Hematemesis associated with a hemoglobin drop was observed in one patient in the SASI group. Upper endoscopy revealed a definite intraluminal gastric source, which was managed by endoscopic clipping. Two patients in the RYGB group presented to the emergency department with acute abdomen and fever. A CT abdomen revealed an intra-abdominal abscess in between bowel loops (one patient was accessible for drainage, while the other improved on antibiotic therapy). Upon reviewing the follow-up US abdomens of both patients, it was observed that the collections had regressed, and their clinical condition had improved. One female patient in the SASI group developed increasing chest pain and dyspnea within 72 h after the operation. A CT scan of the chest revealed signs of a pulmonary embolism prompting urgent treatment with intravenous intensive hydration and systemic anticoagulation. Following treatment, the patient showed improvement, and the embolism resolved without any residual lung damage. A patient in the RYGB group experienced dyspnea because of postoperative chest atelectasis, which was managed by conservative management. An outpatient clinic treated eight patients in both groups for postoperative wound infection and seroma with frequent dressing and drainage.

### Late Complications

In the SASI group, two patients suffered from persistent unresolving vomiting. One patient underwent upper gastrointestinal endoscopy, which revealed a gastro-ileal anastomosis stenosis. A laparoscopic revision of the anastomosis was performed. Another patient developed symptoms of complete bowel obstruction, which did not resolve after conservative treatment and required urgent laparoscopic exploration. A twisted gastro-ileal anastomosis was found and was revised laparoscopically. One patient suffered intractable peripheral neuropathy that made him immobile and totally dependent on a wheelchair. After consulting an internist, a full vitamin assay revealed vitamin B12 deficiency. Parenteral supplementation began with remarkable improvements in pain and overall health. One patient experienced protein-energy malnutrition; this patient presented 6 months after the operation with severe hypoalbuminemia (1.4 g/dl). Intensive nutritional supplements improved his albumin level to 2.5 g/dl, preparing him for revisional surgery by dismantling the gastro-ileal anastomosis. Following gastric specimen extraction, one patient developed an incisional hernia at the trocar site, with a subsequent surgical repair 3 months postoperatively.

There was no significant difference between the two groups as regards postoperative complications (OR = 0.94, 95% CI (0.36–2.47), *p* = 1.000). There were no mortalities in both groups during the follow-up duration.

In the RYGB group, two patients on follow-up visits had anemia with Hb < 9 g/dl, which was improved by parental iron therapy, while four patients suffered from constipation. On follow-up, they were advised to increase fluid and fiber intake and use bulky stool drugs. Except for these patients, there were no other nutritional deficiencies in either group.

### Follow-Up

The outpatient bariatric clinic scheduled follow-up visits for patients at 3, 6, and 12 months postoperatively. All patients in both groups attended their follow-up visits at 3, 6, and 12 months. During the 12-month follow-up, two patients in the SASI group and one patient in the RYGB group were reached by telephone due to traveling overseas, while the remaining patients attended physically in the follow-up clinic.

### Weight Loss Data

**Fig. 1:**
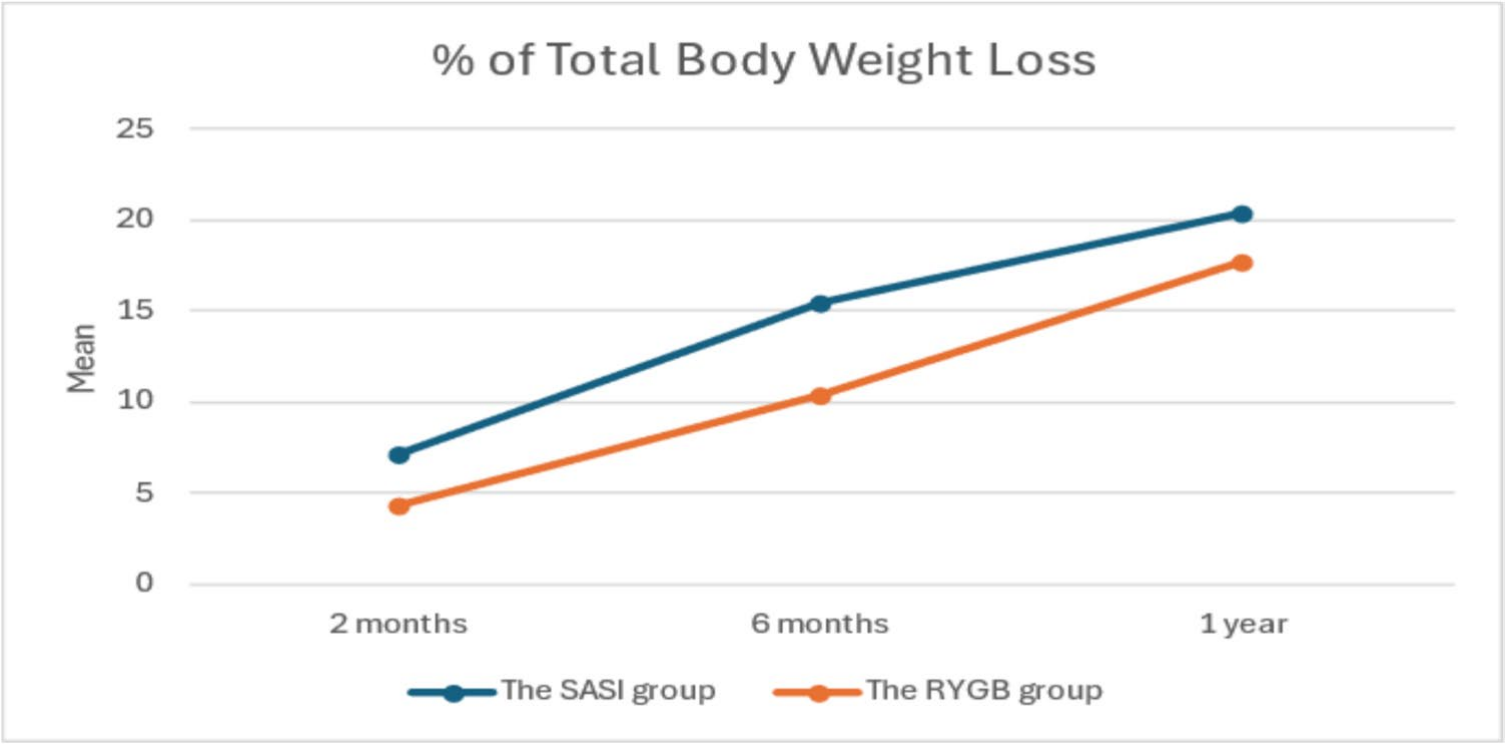
Comparison between the two studied groups according to % TWL

**Fig. 2:**
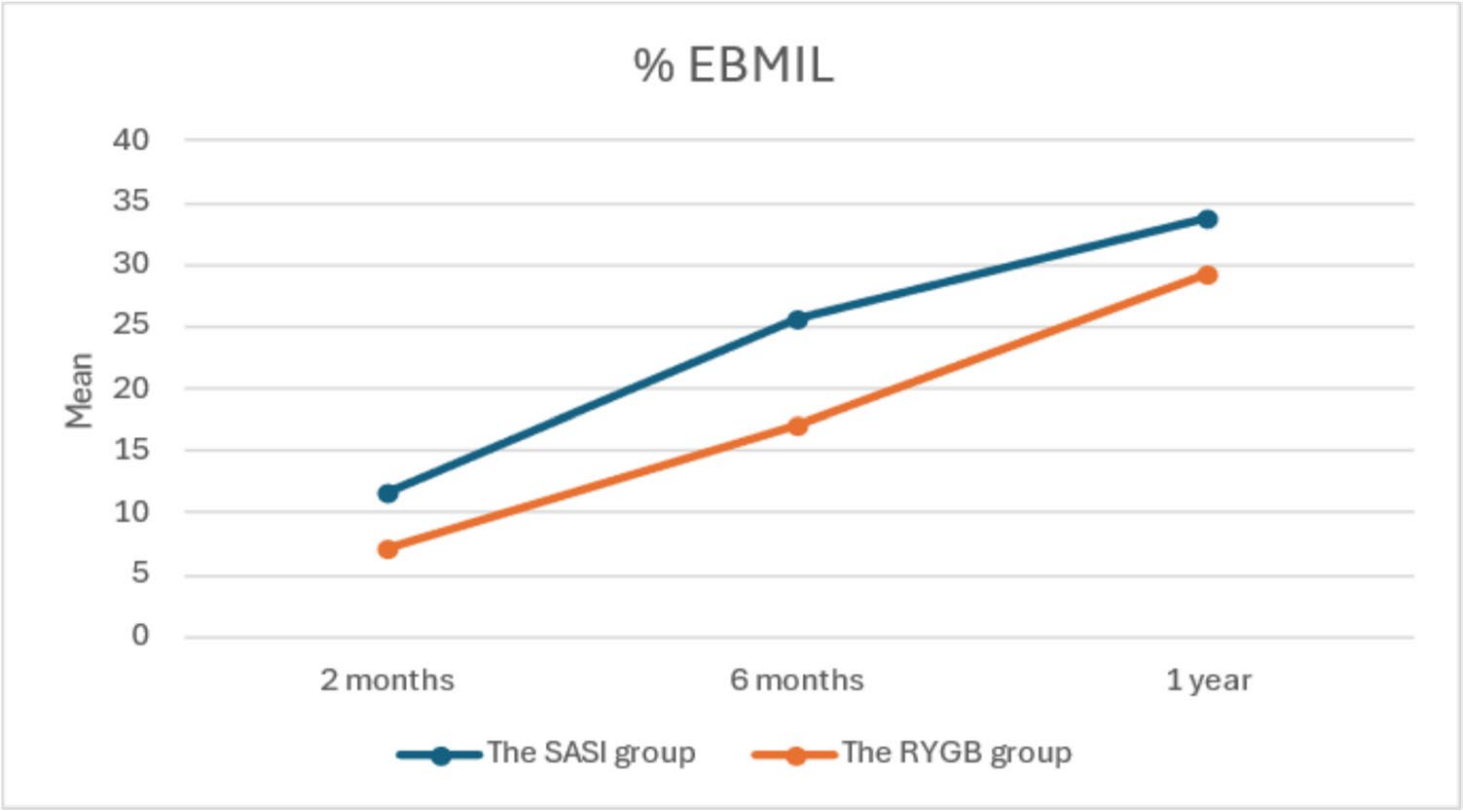
Comparison between the two studied groups according to % EBMIL

Table 5 summarizes the patients’ BMI, %TWL, and % EBMIL throughout the follow-up period, respectively. There was a statistically significant weight loss with time compared to the initial weight in both groups throughout the follow-up period (*p* value of both groups ≤ 0.001). The SASI group showed a significant earlier weight reduction at 3.6 months postoperatively, with no significant weight reduction compared to RYGB at the 1-year follow-up. Starting from the third month postoperatively, there was a statistically significant decrease in both BMI and %TWL in both groups (*p* value of both groups ≤ 0.001). During follow-up periods, there was a statistically significant increase in the percentage of excess BMI loss in both groups; %EBMIL and % TWL were higher in the SASI group (Figs. 1 and 2), but there was no statistically significant difference in %EBMIL between the two groups.

**Table 5:** Comparison between two studied groups according to BMI, % TWL, and %EBMIL at follow-up periods

		Pre	Follow-up			*F*	*p*_3_
			3 month	6 months	1 year		
**BMI**	**SASI group (*n* = 33)**						
	Min.–Max	60.94–66.41	51.56–61.73	43.75–60.20	39.06–58.68	140.413^*^	< 0.001^*^
	Mean ± SD	63.31 ± 1.63	58.83 ± 2.48	53.44 ± 4.83	50.33 ± 5.96		
	***p***_**1**_		< 0.001^*^	< 0.001^*^	< 0.001^*^		
	**RYGB group (*n* = 40)**						
	Min.–Max	60.21–69.33	57.77–64.94	51.56–61.67	45.17–58.10	690.518^*^	< 0.001^*^
	Mean ± SD	63.48 ± 2.70	60.68 ± 2.30	56.89 ± 2.84	52.26 ± 3.89		
	*d* (95%CI)	0.07 (− 0.39–0.54)	0.78 (0.29–1.25)	0.89 (0.41–1.38)	0.39 (− 0.07–0.86)		
	***p***_**1**_		< 0.001^*^	< 0.001^*^	< 0.001^*^		
	***t* (*p***_**2**_**)**	0.315(0.754)	3.314 (0.001)	3.801 (0.001)	1.662 (0.101)		
**% TWL**	**SASI (*n* = 33)**						
	Min.–Max		4.71–17.96	7.06–34.12	9.41–41.18	83.728^*^	< 0.001^*^
	Mean ± SD		7.08 ± 3.06	15.53 ± 7.79	20.44 ± 18.01		
	***p***_**1**_			< 0.001^*^	< 0.001^*^		
	**RYGB (*n* = 40)**						
	Min.–Max		2.47–8.33	6.17–16.97	11.60–27.27	372.073^*^	< 0.001^*^
	Mean ± SD		4.37 ± 1.6	10.35 ± 2.86	17.71 ± 4.35		
	*d* (95%CI)		− 1.14 (− 1.6–0.65)	− 0.92 (− 1.4–0.43)	− 0.22 (− 0.7–0.24)		
	***p***_**1**_			< 0.001^*^	< 0.001^*^		
	***t* (*p*2)**		4.859 (< 0.001)	3.899 (< 0.001)	1.607 (0.112)		
**%EBMIL**	**SASI (*n* = 33)**						
	Min.–Max		7.66–29.83	11.50–54.72	15.33–66.04	86.416^*^	< 0.001^*^
	Mean ± SD		11.71 ± 5.07	25.63 ± 12.59	33.74 ± 15.57		
	***p***_**1**_			< 0.001^*^	< 0.001^*^		
	**RYGB (*n* = 40)**						
	Min.–Max		3.98–13.03	9.96–28.40	18.73–45.65	349.462*	< 0.001^*^
	Mean ± SD		7.19 ± 2.52	17.09 ± 4.65	29.32 ± 7.40		
	*d* (95%CI)		− 1.16 (− 1.66–0.7)	− 0.94 (− 1.4–0.45)	− 0.37 (− 0.84–0.09)		
	***p***_**1**_			< 0.001^*^	< 0.001^*^		
	***t* (*p***_**2**_**)**		4.947 (0.001)	3.976 (0.001)	1.590 (0.116)		

*F F* test (ANOVA), *t* Student’s *t*-test, *BMI* body mass index, *%TWL* percentage of total weight loss, %*EBMIL* percentage of excess body mass index loss, *SD* standard deviation, *d* Cohen’s *d* effect size, *95%CI* 95% confidence interval

*p*_1_: stands for adjusted Bonferroni *p*-value for ANOVA with repeated measures for comparison between preoperative weight and weight at each other period of follow-up

*p*2: comparison between both groups at each period

*p*3: comparison between preoperative condition and at end of follow-up

^*^Statistically significant at *p* ≤ 0.05

A) Obesity-related comorbidities: Table 6 summarizes the amelioration of obesity-related comorbidities. Both operations demonstrated a significant overall resolution of preoperative obesity-related comorbidities. Hypertension was the most frequently encountered condition in the study. 100% hypertension resolution occurred in the RYGB group compared to 83% in the SASI group, with no significant difference between the two groups. Postoperatively, both operations resulted in improvements and resolutions in the diabetes and lipid profiles of the included patients. There were no significant differences between the two groups in terms of improvement in OSAS, reflux symptoms, or the prevalence of musculoskeletal diseases.

**Table 6:** Comparison between the studied groups according to amelioration of obesity-related comorbidities during follow-up

Comorbidities (at follow-up periods)	SASI group (*n* = 33)		RYGB group (*n* = 40)		OR (95%CI)	*p*-value
	No. of patients with comorbidities	% of resolved comorbidity	No of patients with comorbidities	% s of resolved comorbidity		
**OSAS**						
Pre 3 months 6 months 1 year	15 (45.5) 9 5 2	0% 40% 66% 86.7%	12 (30.0) 4 2 1	0% 66.7% 83.3% 91.7%	0.51 (0.19–1.35) 0.29 (0.08–1.07) 0.29 (0.05–1.63) 0.39 (0.03–4.59)	0.225 0.070 0.233 0.586
**Reflux**						
Pre 3 months 6 months 1 year	13 (39.4) 8 6 2	0% 38.5% 53.8% 84.6%	16 (40.0) 2 2 0	0% 87.5% 87.5% 100%	1.03 (0.40–2.63) 0.16 (0.03–0.84) 0.24 (0.04–1.26) –	1.000 **0.036** 0.130 0.201
**HTN**						
Pre 3 months 6 months 1 year	20 (60.6) 10 6 1	0% 50% 70% 95%	28 (70.0) 16 10 0	0% 42.9% 64.3% 100%	1.52 (0.57–4.01) 1.53 (0.58–4.07) 1.50 (0.48–4.68) –	0.462 0.465 0.576 0.452
**DM**						
Pre 3 months 6 months 1 year	9 (27.3) 5 3 1	0% 44.4% 66.7% 88.9%	16 (40.0) 14 12 2	0% 12.5% 25% 87.5%	1.78 (0.66–4.80) 3.02 (0.95–9.54) 4.29 (1.09–16.79) 1.68 (0.15–19.44)	0.324 0.065 **0.041** 1.000
**Dyslipidemia**						
Pre 3 months 6 months 1 year	4 (12.1) 2 1 0	0% 50% 75% 100%	8 (20.0) 8 7 0	0% 0% 12.5% 100%	1.81 (0.49–6.66) 3.88 (0.76–19.71) 6.79 (0.79–58.33) –	0.528 0.101 **0.050** **-**
**Musculoskeletal disease**						
Pre 3 months 6 months 1 year	5 (15.2) 4 0 0	0% 20% 100% 100%	6 (15.0) 4 2 0	0% 33.3% 66.7% 100%	0.99 (0.27–3.58) 0.81 (0.19–3.50) - -	1.000 1.000 0.498 -

All data frequency (percentage)

*p*-value < 0.05 has significant

**Fig. 3:**
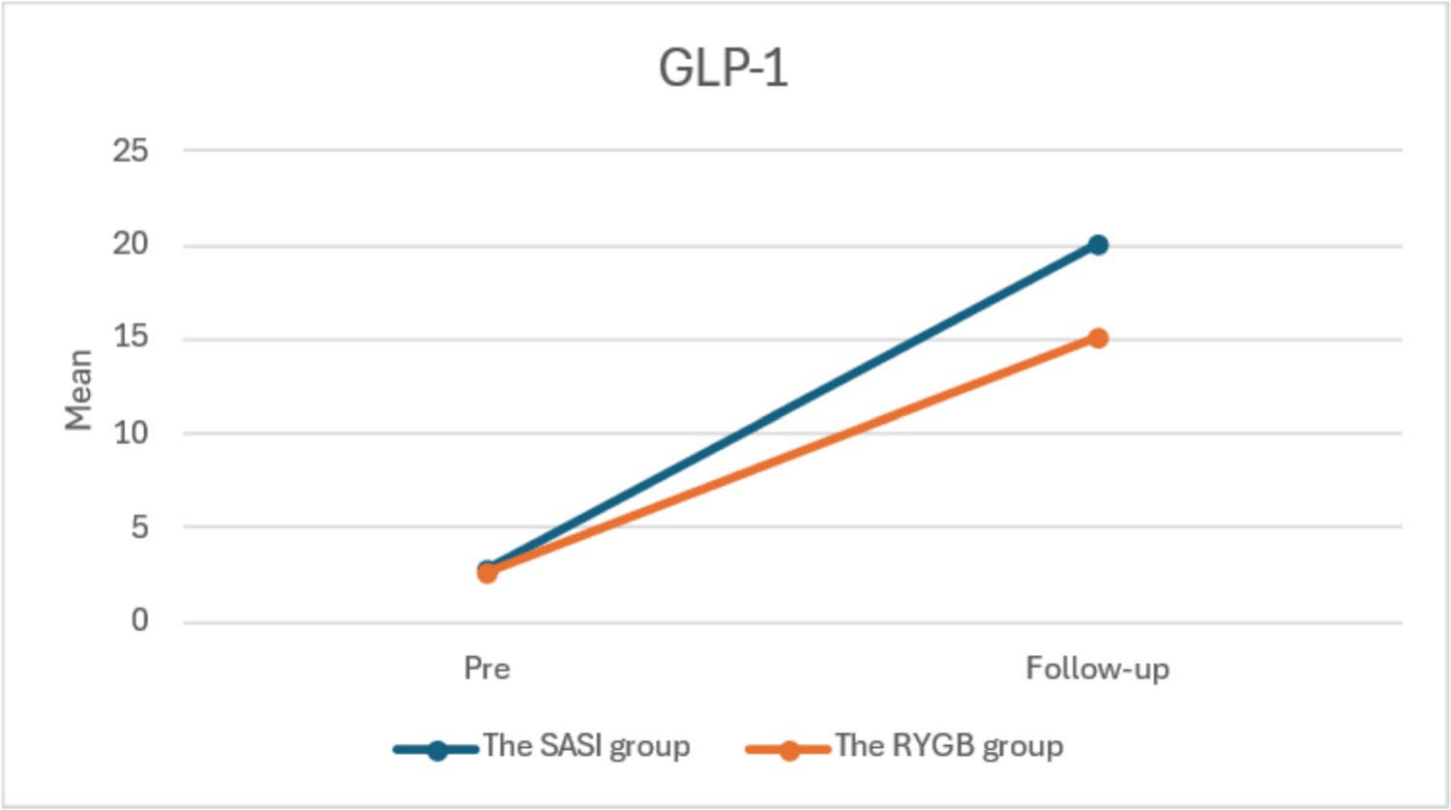
Comparison between the two studied groups according to the level of GLP-1 after 6 weeks

*OR* odds ratio, *95%CI* 95% confidence interval
B) At the end of the follow-up period, the quality of life of the studied patients was assessed, as well as their satisfaction with both operations, using the Moorehead-Ardelt Quality of Life Questionnaire II (M-A QoLQII). There was a significant improvement in quality of life in both groups. The overall results ranged from fair to very good in both groups, which statistically did not result in a significant difference between both groups (*p* value > 0.05).
C) A commercially available radioimmunoassay (glucagon-like peptide (total) RIA Kit; Millipore, Billerica, MA, USA) was used to measure postoperative serum GLP-1 (6 weeks postoperative) and post-prandial GLP-1 (after standard mixed liquid meal challenge by 1 h) (Fig. 3), according to the manufacturer’s instructions. Preoperative postprandial GLP-1 (6 weeks postoperative) was comparable in both groups, with no statistical difference. Following surgery, both groups demonstrated a significant increase in post-prandial GLP-1, with *d* = − 2.58, 95% CI (− 3.2 to − 1.96), *p* < 0.001) with statistically significant higher values in the SASI group, as shown in Table 7.

**Table 7:** Comparison between the studied groups according to the level of GLP-1 preoperative and 6 weeks postoperative

GLP-1 (pmol/l)	Pre	Follow-up	*t*_1_	*p*
**SASI group (*n* = 33)**				
Min.–Max	2.20–4.0	15.60–24.20	38.151^*^	< 0.001^*^
Mean ± SD	2.90 ± 0.42	20.15 ± 2.66		
Median	2.85	19.50		
**RYGB group (*n* = 40)**				
Min.–Max	2.20–3.70	13.0–7.0	65.968^*^	< 0.001^*^
Mean ± SD	2.67 ± 0.41	15.14 ± 1.02		
Median	2.60	15.30		
* d* (95%CI)	− 0.56 (− 1.02–0.1)	− 2.58 (− 3.2–1.96)		
***t***_**2**_** (*p*)**	2.327* (0.023*)	10.190^*^(< 0.001^*^)		

*SD* standard deviation, *d* Cohen’s *d* effect size, *95%CI* 95% confidence interval

*t*_1_: paired *t*-test for comparing between pre and follow-up

*t*_2_: Student’s *t*-test for comparing between SASI and RYGB

^*^Statistically significant at *p* ≤ 0.05

## Discussion

Obesity is a global problem that has unfortunately reached epidemic levels in the past decade [18]. Bariatric procedures achieve weight reduction and subsequent improvement in obesity-related comorbidities by different mechanisms [19]. Gastric restriction and intestinal malabsorption are the two main mechanisms that explain weight loss post-bariatric procedures. Whatever the mechanism, the ideal bariatric operation should be safe for the patients, technically easy and effective in achieving both weight loss and comorbidities resolution [20].

RYGB and SASI bypass are both metabolic surgeries with a completely different mechanism of action. RYGB is the result of several modifications to a gastric bypass operation first described in 1969 by Mason [21], who had observed that postgastrectomy patients with a minor remaining gastric pouch experienced considerable weight reduction. The procedure then divides the stomach into a proximal small pouch and a separate distal large remnant. A gastrojejunal anastomosis then joins the upper pouch to the proximal jejunal bowel loops. Thus, the reduction of the stomach’s storage capacity to nearly 5% of its original preoperative volume allows ingested nutrients to bypass approximately 90–95% of the stomach, the entire duodenum, and a small proximal portion (15–20 cm) of the jejunum. Despite this dual malabsorptive and restrictive mechanism of action, up to 10–15% of patients fail to achieve acceptable weight reduction after the RYGB procedure [22].

With the growing acceptance of the single anastomosis gastric bypass, numerous new techniques emerged aiming to transform a Roux-en-Y bariatric configuration into a simple loop alignment, offering the advantages of a comparatively shorter operative time and a lower risk of internal bowel loop herniation. In this context, Mui et al. [10] reported the first modification of the Santoro procedure into SG with loop bipartition in a case report [10]. This was followed by a case series of 50 patients by Mahdy and Schou [11], with a 1-year follow-up, and then the term single anastomosis sleeve ileal (SASI) bypass was then coined with this study. The idea of weight reduction after the SASI bypass depends mainly on patients stopping eating earlier because of the feeling of a full stomach and due to a hypothalamic-generated satiety sensation initiated by the perception of nutrients when they reach their distal bowel [23]. The “Digestive Adaptation with Intestinal Reserve” (DAIR) research, which primarily focused on the neuroendocrine changes in the gastrointestinal tract, provided the basis for the theory of SASI and SG-TB. The philosophy of this principle involves identifying the physiologic postprandial neuroendocrine response that triggers significant weight loss. The principle of neuroendocrine response has garnered significant attention recently, involving the reduction of proximal gut hormones like ghrelin, resistin, anti-incretins, and gastric inhibitory peptides, thereby facilitating the distal absorption of more nutrients. It leads to more secretion of distal gut hormones such as GLP-1 and peptide YY, which have documented effects on weight loss and T2DM remission [24].

SASI bypass thus powerfully reduces both the ingested meal size and overeating, leading to a decline in animal fat intake by changing taste preferences [11].

Consequently, RYGB and SASI bypass operations possess different mechanisms of action, as the former entails an intestinal segment exclusion with diversion of the ingested meal, whereas the latter adopts the bipartition principle with food switching instead of complete exclusion [25].

Bariatric surgeries in the population of severely obese individuals with BMI ≥ 50 kg/m^2^ and BMI ≥ 60 kg/m^2^ [26], individuals present a challenge for surgeons. To the best of our knowledge, there are no previous studies comparing RYGB and SASI bypass in the treatment of class V obese patients. Therefore, this study was conducted to assess the outcome of the two techniques in terms of both weight loss and improvement of obesity-related comorbidities in class V obese patients.

There were no significant differences between the two procedures with regard to the baseline patients’ characteristics; this minimizes the risk of selection bias in our study, which is retrospective.

Bypass procedures are more complicated as they involve one or more anastomosis of the gastrointestinal tract. In our study, both operations were safe and feasible procedures, with significant differences as regards both, shorter operative time and shorter hospital stays in the SASI group, but both groups showed overall comparable early and late postoperative complications with no significant difference between the two groups. This is comparable to different bariatric studies including the same procedures [3, 11, 23, 27, 28].

The resolution/improvement of obesity-related comorbidities plays an important role in assessing the effectiveness of different bariatric and metabolic surgeries [29]. Weight loss being the primary goal for most patients is closely related to resolution rates of obesity-related comorbidities [28].

Throughout the follow-up period, there was a statistically significant weight reduction with time compared to the initial weights in both groups. The mean PEWL after 3 months was 10.97 ± 5.01 and 6.77 ± 2.51, at 6 months, it was 24.07 ± 12.51 and 16.11 ± 5.01, and finally at 12 months, it was 31.68 ± 15.40 and 27.53 ± 7.57 in the SASI group and the RYGB group, respectively. This is comparable to the study adoped by Mahdy et al. [25], comparing the same procedures although they addressed various obesity categories and adopted a long biliopancreatic limb technique.

Both procedures conferred similar improvements in obesity-related comorbidities with an overall reduction in the number of patients with obesity related comorbidities during a 1-year follow-up.

In the SASI group, 89% DM remission or improvement was achieved. This is comparable to the diabetic profile improvement achieved in the original study adopted by Mahdy and Schou [11], who achieved near 90% improvement after the SASI operation in diabetic profile of their included patients after 1-year follow-up but was lower than that reported in other previous studies [11, 23, 30] that exceeded 95%, with the difference in the targeted population in this study being directed mainly to class V obese patients.

Regarding the improvement in the diabetic profile in the RYGB group, a 87.5% remission or improvement in DM was obtained. This is comparable to a meta-analysis that described a remission or improvement in DM after RYGB was 80% [31].

Several reports have confirmed bariatric surgery’s effectiveness in resolving type 2 diabetes with a diverse remission rate and/or improvement rate. Many studies have proposed predictors of remission of diabetes after weight-loss operations. The majority of these studies included different types of bariatric procedures, particularly RYGB. The explanation for resolution or improvement remains unclear, but decreased energy intake and weight loss probably significantly contribute to this process. Thus, ectopic fat from the hepatic tissue and other stores are mobilized and utilized. A reduction in liver fat content normalizes hepatic insulin sensitivity by improving fasting plasma glucose. Moreover, after 1 week of restricted energy intake, normalization of β-cell function has been demonstrated. Therefore, food restriction can sensibly explain the rapid postoperative metabolic improvement [32–36].

Many studies proposed younger age, shorter duration of DM, lower HbA1c, no preoperative insulin use found a higher rate of complete remission with lower insulin resistance [37, 38].

At 12 months postoperatively, overall hypertension improvement reached 95% versus 100% in the SASI and RYGB groups, respectively. This is slighly better than both; Emile et al. [23] in their study where hypetension improvement after SASI operation reached 54% at 6 months postoperatively, and Zhang et al. [39] in their study where hypetension improvement after RYGB operation reached 32% at 6 months.

Both procedures showed a marvelous improvement in dyslipidemia (100% improvement). This is considered better results for SASI operation in this study compared to dyslipedemia improvement in various studies in the litterarture. SASI operation yielded 65% improvement in dylipidemia in the study adopted by Mahdy et al. [27] and yielded 87.5% improvement in Emile et al. [23] study but showed comparable results to the dyslipedemia improvement after RYGB as regards published studies dealing with the same procedure [25].

The gastro-ileal anastomosis reduces intra-gastric pressure during the SASI bypass procedure, thereby improving preoperative GERD. In our study, reflux improved by 84.5% after SASI, which is comparable to the results of Emile et al. [23], who achieved an 85% reduction in reflux symptoms, and Mahdy et al. [27], who achieved a 90% reduction in reflux symptoms.

In summary, when comparing RYGB and SASI bypass, both operations were associated with significant satisfactory weight loss, comparable postoperative complications, and equivalent resolution or improvement of obesity-related comorbidities with no nutritional complications in both groups throughout the whole follow-up period. The SASI operation had a significantly shorter operative time (skin-to-skin) and postoperative total hospital stay compared to the RYGB operation. However, the elimination of two ways for food passage and one anastomosis decreases nutritional deficiency and the possibility of surgically related complications. Although most procedures reduce postoperative endoscopic access, patients undergoing SASI bypass have excellent endoscopic access to the duodenum and biliary systems.

The primary functions of GLP-1 include the enhancement of glucose-stimulated insulin secretion, beta cell growth and survival, glucagon release inhibition, and food intake control. Current data suggest that hormonal mechanisms rather than weight loss alone account for the beneficial effect of bariatric surgery on T2DM [40]. Both techniques were associated with a significant increase in GLP-1 levels compared with their preoperative values. Like earlier studies [41, 42], GLP-1 levels increased in response to an oral glucose load with both procedures. Furthermore, GLP-1 levels were significantly higher in the SASI group than in the RYGB group 6 weeks postoperatively. The Romero et al. [43] hypothesis, which maintains the normal food pathway and allows the proximal part of the stomach to absorb a small amount of consumed food, explains this. Additionally, the bipartition method appears to have a lower nutritional impact. Combined sleeve gastrectomy causes a decrease in ghrelin while also allowing food to move through the anastomosis quickly, which enhances the distal bowel’s metabolic effects.

In our study, both procedures resulted in a significant rise in six weeks postprandial postoperative GLP-1 levels. The remarkably large effect sizes observed for GLP-1 changes (Cohen’s *d* > 9 for both procedures) are a direct result of the significant postoperative increases relative to low preoperative values, coupled with minimal variability in measurements. These values highlight the clinical relevance of the observed changes rather than serving solely as statistical metrics.

Improved quality of life (QoL) is the ultimate goal of bariatric surgery and one of the most common motivators for patients to seek surgery. Weight loss, diabetes resolution, and other comorbidities seem to significantly improve the quality of life. In this study, both procedures resulted in a satisfactory improvement in quality of life.

## Limitations

This study has several significant shortcomings that need to be addressed. The retrospective design inherently poses a risk of selection and information bias. Retrospective analyses depend on pre-existing data, which may be insufficient or inconsistently documented, potentially compromising the credibility of the results. Despite attempts to address these difficulties, they persist as a worry, highlighting the necessity for future investigations to confirm the findings.

Secondly, the study’s performance at a single center limited the results’ generalizability. Multicenter studies with varied patient populations are essential to validate the external applicability of these findings.

The limited follow-up duration of 12 months offers significant insights into initial results but is inadequate for evaluating long-term effects. Patients may subsequently experience significant weight regain and recurrence of related comorbidities such as diabetes or hypertension, which were not evident during the research period. The aforementioned difficulties are essential for comprehending the durability and safety of the intervention, highlighting the necessity for prolonged follow-up in subsequent investigations.

Rectifying these limitations in further research would augment our comprehension of the intervention’s enduring efficacy, safety, and generalizability, enhancing patient outcomes and guiding therapeutic decision-making.

## Conclusion

In populations with class v obesity, the SASI procedure had a statistically shorter operative time and a shorter hospital stay compared to RYGB. Both procedures resulted in satisfactory weight loss as well as comparable improvements in obesity-related comorbidities. SASI procedures had statistically significant increase in postoperative GLP-1 (6 weeks postoperative) which had a good impact on resolution of type 2 DM. In future research, the need for controlled prospective, multicenter studies with larger patient cohorts and longer follow-up periods to better assess the long-term outcomes of SASI versus RYGB inpatients with class V obesity.

## Data Availability

No datasets were generated or analysed during the current study.

## References

[1] WHO Consultation on Obesity (1999: Geneva, Switzerland) & World Health Organization. Obesity: preventing and managing the global epidemic: report of a WHO consultation. World Health Organization. 2000. https://iris.who.int/handle/10665/42330. 11234459

[2] Guh DP, Zhang W, Bansback N, et al. The incidence of co-morbidities related to obesity and overweight: a systematic review and meta-analysis. BMC Public Health. 2009;9:1–20. 19320986 10.1186/1471-2458-9-88 PMC2667420

[3] Nasser H, Ivanics T, Leonard-Murali S, et al. Perioperative outcomes of laparoscopic Roux-en-Y gastric bypass and sleeve gastrectomy in super-obese and super-super-obese patients: a national database analysis. Surg Obes Relat Dis. 2019;15(10):1696–703. 31530452 10.1016/j.soard.2019.07.026

[4] Soong T-C, Lee M-H, Lee W-J, et al. Long-term efficacy of bariatric surgery for the treatment of super-obesity: comparison of SG, RYGB, and OAGB. Obes Surg. 2021;31:3391–9. 33993423 10.1007/s11695-021-05464-0

[5] Regan J, Inabnet W, Gagner M, et al. Early experience with two-stage laparoscopic Roux-en-Y gastric bypass as an alternative in the super-super obese patient. Obes Surg. 2003;13(6):861–4. 14738671 10.1381/096089203322618669

[6] Peterson K, Anderson J, Boundy E, et al. Rapid evidence review of bariatric surgery in super obesity (BMI ≥ 50 kg/m(2)). J Gen Intern Med. 2017;32(Suppl 1):56–64. 28271426 10.1007/s11606-016-3950-5 PMC5359153

[7] Yang N, Hua H, Liu S, et al. The current status and challenges of perioperative management of patients with a BMI of greater than or equal to 50 kg/m2 undergoing bariatric surgery in China: a multicenter cross-sectional study. Int J Surg. 2024;110(5):2577–82. 38265423 10.1097/JS9.0000000000001108 PMC11093425

[8] Rubino F, Schauer PR, Kaplan LM, et al. Metabolic surgery to treat type 2 diabetes: clinical outcomes and mechanisms of action. Annu Rev Med. 2010;61(1):393–411. 20059345 10.1146/annurev.med.051308.105148

[9] Azevedo FR, Santoro S, Correa-Giannella ML, et al. A prospective randomized controlled trial of the metabolic effects of sleeve gastrectomy with transit bipartition. Obes Surg. 2018;28:3012–9. 29704228 10.1007/s11695-018-3239-3

[10] Mui WM, Lee WH, Lam KK. Laparoscopic sleeve gastrectomy with loop bipartition: a novel metabolic operation in treating obese type II diabetes mellitus. Int J Surg Case Rep. 2014;5(2):56–8. 24441436 10.1016/j.ijscr.2013.12.002 PMC3921657

[11] Mahdy T, Schou C. Efficacy of single anastomosis sleeve ileal (SASI) bypass for type-2 diabetic morbid obese patients: gastric bipartition, a novel metabolic surgery procedure: a retrospective cohort study. Int J Surg. 2016;34:28–34. 27545956 10.1016/j.ijsu.2016.08.018

[12] Vennapusa A, Panchangam BR, Madivada MS. A feasibility study of novel “laparoscopic sleeve gastrectomy with loop gastroileal bypass” for obesity: an Indian experience. Int Surg. 2017;102(11–12):504–13.

[13] Erol MF, Kayaoglu HA. Comparison of the effectiveness of single anastomosis sleeve ileal bypass and Roux-en-Y gastric bypass in obese patients with type 2 diabetes. Obes Surg. 2024;34(10):3748–54. 39162962 10.1007/s11695-024-07472-2

[14] Ataya K, Patel N, Aljaafreh A, et al. Outcomes of single anastomosis sleeve ileal (SASI) bypass as an alternative procedure in treating obesity: an updated systematic review and meta-analysis. Obes Surg. 2024;34(9):3285–97. 39060638 10.1007/s11695-024-07366-3

[15] Felipe LA, Bachi ALL, Oliveira MC, et al. Effects of Roux-en-Y gastric bypass on the metabolic profile and systemic inflammatory status of women with metabolic syndrome: randomized controlled clinical trial. Diabetol Metab Syndr. 2023;15(1):19. 36788619 10.1186/s13098-023-00986-2 PMC9930348

[16] Brethauer SA, Kim J, El Chaar M, et al. Standardized outcomes reporting in metabolic and bariatric surgery. Obes Surg. 2015;25:587–606. 25802064 10.1007/s11695-015-1645-3

[17] Avsar FM, Ozel H, Topaloglu S, et al. Improvement of vertical banded gastroplasty by strict dietary management. Obes Surg. 2004;14(2):265–70. 15018759 10.1381/096089204322857681

[18] Mitchell S, Shaw D. The worldwide epidemic of female obesity. Best Pract Res Clin Obstet Gynaecol. 2015;29(3):289–99. 25487257 10.1016/j.bpobgyn.2014.10.002

[19] Mulla CM, Middelbeek RJ, Patti ME. Mechanisms of weight loss and improved metabolism following bariatric surgery. Ann N Y Acad Sci. 2018;1411(1):53–64. 28868615 10.1111/nyas.13409 PMC5788713

[20] Rutledge R. The mini-gastric bypass: experience with the first 1,274 cases. Obes Surg. 2001;11(3):276–80. 11433900 10.1381/096089201321336584

[21] Mason EE, Ito C. Gastric bypass. Ann Surg. 1969;170(3):329–39. 5804373 10.1097/00000658-196909010-00003 PMC1387676

[22] Elnahas AI, Jackson TD, Hong D. Management of failed laparoscopic Roux-en-Y gastric bypass. Bariatr Surg Pract Patient Care. 2014;9(1):36–40. 24761371 10.1089/bari.2013.0012 PMC3963694

[23] Emile SH, Madyan A, Mahdy T, et al. Single anastomosis sleeve ileal (SASI) bypass versus sleeve gastrectomy: a case-matched multicenter study. Surg Endosc. 2021;35:652–60. 32072282 10.1007/s00464-020-07430-w

[24] Mahdy T, Gado W, Emile S. Single anastomosis sleeve ileal (SASI) bipartition. In: Agrawal S, editor. Obesity, bariatric and metabolic surgery: a comprehensive guide. Cham: Springer International Publishing; 2023. p. 867–81.

[25] Mahdy T, Emile SH, Alwahedi A, et al. Roux-en-Y gastric bypass with long biliopancreatic limb compared to single anastomosis sleeve ileal (SASI) bypass in treatment of morbid obesity. Obes Surg. 2021;31(8):3615–22. 33942216 10.1007/s11695-021-05457-z

[26] Renquist K. Obesity classification. Obes Surg. 1998;8(4):480. 9731686 10.1381/096089298765554403

[27] Mahdy T, Emile SH, Madyan A, et al. Evaluation of the efficacy of single anastomosis sleeve ileal (SASI) bypass for patients with morbid obesity: a multicenter study. Obes Surg. 2020;30:837–45. 31734889 10.1007/s11695-019-04296-3

[28] Sugerman HJ, editor Gastric bypass surgery for severe obesity. Seminars in Laparoscopic Surgery; 2002: Sage Publications Sage CA: Thousand Oaks, CA. 12152150

[29] Wang Y, Song Y-H, Chen J, et al. Roux-en-Y gastric bypass versus sleeve gastrectomy for super super obese and super obese: systematic review and meta-analysis of weight results, comorbidity resolution. Obes Surg. 2019;29:1954–64. 30953336 10.1007/s11695-019-03817-4

[30] Salama TM, Sabry K, Ghamrini YE. Single anastomosis sleeve ileal bypass: new step in the evolution of bariatric surgeries. J Invest Surg. 2017;30(5):291–6. 27768400 10.1080/08941939.2016.1241841

[31] Buchwald H, Avidor Y, Braunwald E, et al. Bariatric surgery: a systematic review and meta-analysis. JAMA. 2004;292(14):1724–37. 15479938 10.1001/jama.292.14.1724

[32] Praveen Raj P, Bhattacharya S, Saravana Kumar S, et al. Do bariatric surgery-related type 2 diabetes remission predictors add clinical value? A study on Asian Indian obese diabetics. Obes Surg. 2017;27(8):2113–9. 28236254 10.1007/s11695-017-2615-8

[33] Robert M, Ferrand-Gaillard C, Disse E, et al. Predictive factors of type 2 diabetes remission 1 year after bariatric surgery: impact of surgical techniques. Obes Surg. 2013;23(6):770–5. 23355293 10.1007/s11695-013-0868-4

[34] Souteiro P, Belo S, Neves JS, et al. Preoperative beta cell function is predictive of diabetes remission after bariatric surgery. Obes Surg. 2017;27(2):288–94. 27435450 10.1007/s11695-016-2300-3

[35] Knop FK, Taylor R. Mechanism of metabolic advantages after bariatric surgery: it’s all gastrointestinal factors versus it’s all food restriction. Diabetes Care. 2013;36(Suppl 2):S287-91. 23882061 10.2337/dcS13-2032 PMC3920787

[36] Lim EL, Hollingsworth KG, Aribisala BS, et al. Reversal of type 2 diabetes: normalisation of beta cell function in association with decreased pancreas and liver triacylglycerol. Diabetologia. 2011;54(10):2506–14. 21656330 10.1007/s00125-011-2204-7 PMC3168743

[37] Scopinaro N, Adami GF, Bruzzi P, et al. Prediction of diabetes remission at long term following biliopancreatic diversion. Obes Surg. 2017;27(7):1705–8. 28101844 10.1007/s11695-017-2555-3

[38] Bhasker AG, Remedios C, Batra P, et al. Predictors of remission of T2DM and metabolic effects after laparoscopic Roux-en-y gastric bypass in obese Indian diabetics-a 5-year study. Obes Surg. 2015;25(7):1191–7. 25399348 10.1007/s11695-014-1501-x

[39] Zhang N, Maffei A, Cerabona T, et al. Reduction in obesity-related comorbidities: is gastric bypass better than sleeve gastrectomy? Surg Endosc. 2013;27:1273–80. 23239292 10.1007/s00464-012-2595-7

[40] Ochner C, Gibson C, Shanik M, et al. Changes in neurohormonal gut peptides following bariatric surgery. Int J Obes. 2011;35(2):153–66. 10.1038/ijo.2010.132 PMC3632050 20625384

[41] Abdalaziz A, Sarhan MD, Abou-Eisha HA, et al. Laparoscopic single anastomosis sleeve ileal bypass with follow-up of weight loss and metabolic impact. Open Access Macedonian J Med Sci. 2022;10(B):1325–31.

[42] Arble DM, Evers SS, Bozadjieva N, et al. Metabolic comparison of one-anastomosis gastric bypass, single-anastomosis duodenal-switch, Roux-en-Y gastric bypass, and vertical sleeve gastrectomy in rat. Surg Obes Relat Dis. 2018;14(12):1857–67. 30292648 10.1016/j.soard.2018.08.019 PMC6294714

[43] Romero RJ, Colorado-Subizar R, De Uriarte-Lorente M, et al. Single anastomosis sleeve ileal bypass (SASI Bypass): short-term outcomes and concerns. Obes Surg. 2021;31(5):2339–43. 33405181 10.1007/s11695-020-05145-4

